# Extracellular Vesicle Flow Cytometry Analysis and Standardization

**DOI:** 10.3389/fcell.2017.00078

**Published:** 2017-08-30

**Authors:** Joshua A. Welsh, Judith A. Holloway, James S. Wilkinson, Nicola A. Englyst

**Affiliations:** ^1^Faculty of Medicine, University of Southampton Southampton, United Kingdom; ^2^Optoelectronics Research Centre, University of Southampton Southampton, United Kingdom

**Keywords:** extracellular vesicles, EV, Extracellular vesicles (EVs), flow cytometry (FCM), scattering, fluorescence standardization, scattering standardization

## Abstract

The term extracellular vesicles (EVs) describes membranous vesicles derived from cells, ranging in diameter from 30 to 1,000 nm with the majority thought to be in the region of 100–150 nm. Due to their small diameter and complex and variable composition, conventional techniques have struggled to accurately count and phenotype EVs. Currently, EV characterization using high-resolution flow cytometry is the most promising method when compared to other currently available techniques, due to it being a high-throughput, single particle, multi-parameter analysis technique capable of analyzing a large range of particle diameters. Whilst high resolution flow cytometry promises detection of the full EV diameter range, standardization of light scattering and fluorescence data between different flow cytometers remains an problem. In this mini review, we will discuss the advances in high-resolution flow cytometry development and future direction of EV scatter and fluorescence standardization. Standardization and therefore reproducibility between research groups and instrumentation is lacking, hindering the validation of EVs use as diagnostic biomarkers and therapeutics.

## Introduction

The term “extracellular vesicles” (EVs) refers to membranous vesicles derived from cells. Types of EV include exosomes (30–150 nm) and microvesicles (30–1,000 nm). EVs have shown potential as translational biomarkers and therapeutics, as well as developing our knowledge of intercellular communication (van der Pol et al., 2012a; Barteneva et al., 2013; Buzas et al., 2014; Colombo et al., 2014; Fleury et al., 2014). To date the derivation and potential function of many circulating EVs has been inferred by the expression of surface proteins. Currently however, EV analysis is hindered by the resolution of commercially available phenotyping instrumentation and the lack of standardization between these pieces of equipment.

EVs have been reported to be associated with a wide range of conditions including: cancer, autoimmune disease, blood disorders, cardiovascular disease, infectious disease, metabolic disease, and more (Barteneva et al., 2013). Not only have EVs shown promise as diagnostic and prognostic biomarkers, they have also shown potential as therapeutics (Gyorgy et al., 2015). In the majority of studies conducted to date the association between numbers and phenotype of EVs and a clinical condition has relied upon phenotyping EVs through surface protein expression, allowing the cell derivation and insight into EV function to be deduced (Barteneva et al., 2013). However, these studies have also been hampered in their ability to fully deduce EV phenotypes and therefore gain deep insight into EV modulation due to the sensitivity limits of current instrumentation. This is due to the majority of EVs expressing only tens of copies of a protein due to their small diameter, unlike cells which express thousands of copies (Gardiner et al., 2013; Varga et al., 2014; Familari et al., 2015; Nolan, 2015; Alberro et al., 2016). Even when these few proteins are labeled with the brightest of fluorescently-conjugated antibodies, they are below the limit of detection of most commercially available equipment.

Due to this limitation in sensitivity, translational studies conducted to date have been limited, particularly for the majority of EVs with low expressing protein markers. An EV surface protein may have been thought to be unexpressed due to being undetectable on current equipment, when in fact the sensitivity of the instrument was just not high enough to detect the protein expression. Furthermore, many translational studies may have only been making associations based on the minority of vesicles, due to the smaller majority of vesicles being beyond the detection limits of the instrument. The validity of studies making associations based on the minority of the EV population is therefore questionable. These issues are compounded by the lack of standardization methods implemented and reporting that makes it impossible to know what proportion of the EV population was phenotyped or counted, or what the minimum number of proteins detectable was on an instrument. These issues are in part due to lack of reference standards available, lack of guidelines, and lack of instrumentation sensitive enough to detect and phenotype extracellular vesicles.

The ideal EV phenotypic analysis instrumentation characteristics would include: determination of EV diameter, accurate particle concentration determination, single particle analysis, multi-parameter phenotyping to allow identification of different types of particles through multiple antigens, relatively high-throughput, with all of the above for the full range of EVs (30–1,000 nm) without alterations to the equipment e.g., doesn't require physical instrumentation alterations for certain EV diameter ranges. A wide variety of equipment has been used for EV analysis but currently no single commercial instrument meets these criteria.

Instrumentation commonly utilized to date include electron microscopy (EM), conventional flow cytometry, nanoparticle tracking analysis (NTA), dynamic light scatter (DLS), and resistive pulse sensing (RPS). An overview of how these EV analysis instruments and others compare in terms of detection characteristics and sample preparation characteristics is given in Table 1. While a number of existing techniques are useful for single EV diameter or concentration determination, such as EM, NTA, RPS, they are limited in their ability to provide multi-parameter phenotypes, Table 1. Dedicated flow cytometry however, offers the ability for multi-parameter single particle analysis for EVs. Further detailed reviews and comparisons of alternative analyses equipment can found in the literature (van der Pol et al., 2010, 2013; Rupert et al., 2017).

**Table 1:** A comparison of currently utilized EV detection equipment properties.

**Technique**	**Diameter determination**	**Resolve full EV diameter range**	**Heterogeneous diameter differentiation**	**EV concentration determination**	**EV phenotype determination**	**Multi-parametric phenotyping**	**High-throughput analysis**	**Non-destructive sample preparation**
dFCM	+	+	+	+	+	+	+	+
cFCM	+	−	+	+	+	+	+	+
AFM	+	+	+	−	−	−	−	+
EM	+	+	+	+	+	−	−	−
SRM	+	+	+	+	+	+	−	+
RPS	+	−	+	+	−	−	+	+
DLS	+	−	−	+	−	−	+	+
NTA	+	−	+	+	+	−	+	+

The technique showing the most promise in meeting all criteria for ideal EV analysis is flow cytometry instrumentation that's had it components optimized solely for small particle analysis; dedicated flow cytometry. Conventional flow cytometry resolution has steadily improved alongside dedicated flow cytometry resolution due to technological advancements in several areas that have benefitted its fundamental components. Due to these improvements EV detection using conventional flow cytometry has become possible, and is currently estimated to be utilized in 90% of EV research (Valkonen et al., 2016). Because conventional flow cytometry has made EV detection possible to a degree, and dedicated flow cytometer are becoming available, it has become apparent that the diversity in sample preparation, instrumentation, and instrument detection settings, have a large impact on the comparability of EV data between research groups and equipment (Lacroix et al., 2010, 2013). EV sample variably due to sample preparation will not be discussed here, but is discussed at length in the literature (Chandler, 2013; Boing et al., 2014; Cvjetkovic et al., 2014; Kormelink et al., 2015; Szatanek et al., 2015; Gardiner et al., 2016; Pol et al., 2016).

## Detection of EVs using flow cytometry

**Figure 1:**
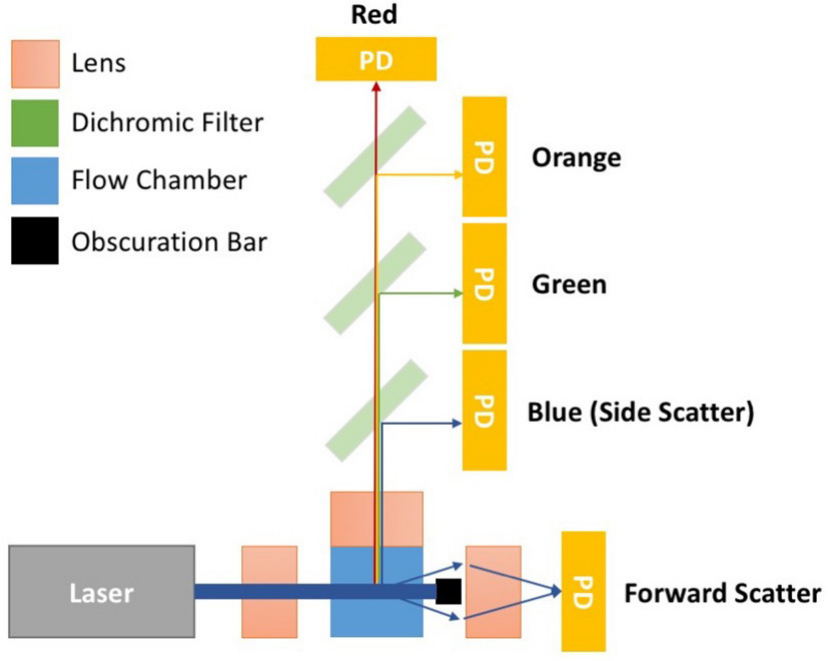
Simplified flow cytometer layout. The top down view of the flow cytometry components depicts a monochromatic interrogation laser beam traversing through air before being focussed by a lens onto the silica flow chamber. Within the flow chamber is a hydrodynamically focussed core stream. The illuminated region of the core stream, where particles are passed through the beam, is known as the interrogation zone. Light scattered from the particles is sent in all directions. The light scattered perpendicular to the laser beam is focussed onto detectors by an optically coupled collection lens. This light then travel through a series of filters to split the light wavelength before reaching a photon detector (PD). PDs will convert photons to electrical signals will be sent to a digital acquisition (DAQ) board for further analysis. The forward scattered light is collected from around the obscuration bar, which stops the laser beam light saturating the forward scatter detector. Points 1–4 outline some of the changes to conventional flow cytometry that would create a dedicated small particle flow cytometer. (1) Lasers—high powered lasers, with a smaller focussed beam spot size, (2) Fluidics—a stable slow velocity core stream, that has a small diameter (1–2 μm), (3) Fluorescence/Side scatter optics—smaller fluorescence/side scatter collection optical apertures and/or higher sensitivity detectors e.g., avalanche photodiodes, (4) Forward scatter optics—larger forward scatter obscuration bars and higher sensitivity detectors.

Flow cytometers are comprised of three integral systems: fluidics, optics, and electronics. In conventional flow cytometry a sample is drawn up, before being suspended in a hydrodynamically focused fluid stream (the fluidics system) that is passed through a series of laser beams where the sample's particles are illuminated. Upon illumination, a particle's scattered and fluorescent light from particles is collected perpendicular to the incident laser beam (optical system). Forward scattered light is also collected at a small angle to the laser beam incident angle. Scattered and fluorescent light is fed to photodetectors and signals are further processed by electronics (electronics system). For an event to be recorded using flow cytometry it must exceed a triggering threshold. The triggering threshold is generally set on forward scatter for cells, set at a level that is below the power of cells scattered light but above the systems background noise. An overview of a simplified flow cytometer layout is given in Figure 1. A dedicated small particle flow cytometer would typically differ from a conventional flow cytometry by having: (1) high powered lasers, with a smaller focussed beam spot size, (2) a stable slow velocity core stream with a small diameter (1–2 μm), (3) smaller fluorescence/side scatter collection optical apertures and/or higher sensitivity detectors e.g., avalanche photodiodes, (4) larger forward scatter obscuration bars and higher sensitivity detectors, Figure 1. Flow cytometer measurements are based around the quantification of both scattered and fluorescent light reaching these detectors and it is the calibration of these optical signals and standardization between instruments that is required for EV analysis using flow cytometry.

## Scattered light

When light reaches a particle, light is scattered, and the term “scattering” encompasses diffraction, reflection, refraction, and absorption and re-radiation (van de Hulst, 1981). From here on in, the term “scattering” shall refer to the combined effects of all these phenomena. Flow cytometers detect scatter using optical filters that allow light at the illumination wavelength to pass into the detector, commonly this is at 488 nm. Scatter measurements are conventionally taken in the forward direction (forward scatter; FSC) and perpendicular to (side scatter; SSC) to illuminating laser. Factors affecting how much light a particle scatters are: the illuminating wavelength, the suspending medium refractive index, and the particle's diameter and refractive index.

## Scatter detection standardization

The relationship between different particles measured scatter is heavily influenced by range of collection angles a flow cytometer's scatter detector receives. The scattering collection angle range varies considerably between flow cytometers, which has led to inconsistencies in the reporting of EV data and bead reference standards (van der Pol et al., 2012a). The relevance of this variation to EV analysis was first identified by Van der Pol. A method to account for this variation and to standardize scattering measurements was also put forward utilizing light scatter modeling based on Mie theory. This approach allowed comparison of data recorded on instruments with differing light scatter collection configurations. This method of standardization is however initially complex to set up due to knowledge in coding, light scatter physics and mathematics being required to implement. Furthermore, access to proprietary flow cytometer component information is required to determine the limiting collection angle range of the instrument. Once implemented however, this method of standardization only requires beads of known diameter and refractive index to be analyzed on an instrument at the same scatter detector voltages used for the EV samples being analyzed.

Scatter models exhibit a very high correlation between the predicted scatter intensity of beads with known diameter and refractive index, and the acquired scattered power using a flow cytometer (van der Pol et al., 2012a). Limitations arise however when translating this to EV standardization where an EV refractive index has to be assumed in order to determine particle diameter. The refractive index of an EV depends on the ratio of cytosol to membrane, the amount and type of cargo contained within the cytosol, and the number and type of proteins expressed on the membrane. Currently, EV refractive indices have been inferred from determining the refractive index of cellular components, or calculated using nanoparticle tracking analysis. Recently, a form of dedicated flow cytometry that collects particle light scattering from a number of collection angles has also enabled RI determination of particles (Maltsev, 2000; van der Pol et al., 2012b, 2014; Gardiner et al., 2014; Konokhova et al., 2016). From the limited studies to date on EV refractive index, it has been demonstrated that EVs derived from different cells show variation between their refractive indices, which is most likely due to the types of proteins expressed and intracellular cargo differences between cells (Gardiner et al., 2014).

Whilst scatter standardization currently relies upon theoretical modeling combined with the analysis of beads of known diameter and refractive index, the availability of reference particles with refractive index close to that of EVs for standardization would yield accurate EV diameter data directly, without the need for complex models. These types of particles would be useful in quickly determining whether a flow cytometer's EV scatter resolution is suitable for EV analysis without the need for modeling. Deciding on the best refractive index for such particles and validating their use across different EV derivations would however be required.

The use of scatter modeling as a method of standardization, whilst not yet fully validated, has been pivotal in demonstrating the need for the development of better diameter standards in the field, and has emphasized why EV diameter should not simply inferred to be equal to the diameter of a detected polystyrene or silica particle population at the same scatter intensity, as it has in the past (van der Pol et al., 2012b).

## Fluorescent light

Fluorescence is utilized in flow cytometry, and many other techniques, in a variety of ways such as identifying membrane bound proteins through binding of fluorescently-conjugated antibodies, membrane staining using fluorescent dyes, and intracellular content labeling using fluorescent probes. Fluorescence is a three-stage process: excitation, lifetime in an excited-state lifetime and fluorescence emission (Shapiro, 2003). The amount of fluorescence light a flow cytometer's detector receives is dependent on the fluorophore's emitted power (brightness), the number of fluorescent molecules being illuminated, the flow cytometer's laser intensity, along with the characteristics of the optical components between the fluorophore and the detector (Schwartz et al., 2004). Fluorescence intensity can be quantified in terms of standard units known as molecule of equivalent soluble fluorophore (MESF), determined by comparing the fluorescence intensity signal from a bead standard to the signal from a solution of the same fluorophore (Schwartz et al., 2002; Gaigalas et al., 2005).

## Fluorescence detection standardization

It has been demonstrated that enumeration of EVs using a fluorescent triggering threshold, employing a fluorescent membrane intercalating dye, offers higher sensitivity than using scatter triggering threshold (Nolte-'t Hoen et al., 2012; van der Vlist et al., 2012; Arraud et al., 2015a,b; Pasalic et al., 2016; Stoner et al., 2016). This method of detection requires a wash step, or titering of the dye, to ensure minimal quantities of residual dye. This ensures that a high signal to noise ratio is obtained, allowing more sensitive and accurate detection of positive stained EVs. Using this technique, some conventional flow cytometers are capable of detecting the full EV diameter range (Stoner et al., 2016). However, in samples where there may be high lipoprotein content, differentiating EVs from lipoproteins would require sufficient scatter resolution to separate the high refractive index lipoproteins from the lower refractive index EVs.

Despite there being cases where the whole EV range is detectable by membrane fluorescence on conventional flow cytometers, these instruments are not capable of determining the protein expression of smaller EVs. Cell proteins that are highly expressed on the parent cell may only be present at <100 copies for the majority of their derived vesicles, due to protein expression being limited by the EVs' surface area (Nolan, 2015). Flow cytometers therefore need to be able to detect very few fluorescent molecules, down to single fluorescent molecules, if the full range of EVs is to be phenotyped. While there are some challenges in developing this capability into a commercial flow cytometry, single molecule detection by fluorescence was demonstrated as early as the 1980s, with detection capabilities today sensitive to less than a single fluorescent molecule (Nguyen et al., 1987; Zhu et al., 2014).

Currently, conventional flow cytometers are not capable of detecting single fluorescent molecules. When fluorescently-labeled antibodies are used to stain EV surface proteins, larger EVs expressing multiple copies of the antigen of interest will separate from the auto-fluorescent population. As EVs decrease in diameter along with the surface area limited expression of the antigen, the fluorescently positive population will decrease, eventually merging with the auto-fluorescent (natural fluorescence from molecules found in the cell membrane or intracellularly e.g., flavins) population. In this case, the number of fluorescently stained EVs reported will be limited by the analysis instrument fluorescence sensitivity, therefore affecting absolute EV counts. There may however be cases where the fluorescent resolution of a flow cytometer is not sufficient to separate dimly fluorescent EVs from negatively stained EVs. This could either be due to lack of protein surface expression, the use of a dim fluorophore, or a combination of the two. It is therefore important to be able to quantify the fluorescent resolution of flow cytometers, and report the number of fluorescently positive events above a quantified limit of resolution. This could potentially enable comparisons between results from low resolution and high-resolution instruments. Fluorescence calibration and resolution is a relatively well-defined area in flow cytometry with many widely-accepted methods of quantification, that are discussed in detail elsewhere (Steen, 1992; Chase and Hoffman, 1998; Wood, 1998; Wood and Hoffman, 1998; Schwartz et al., 2002, 2004; Graves et al., 2005; Hoffman, 2005; Kantor et al., 2016). Quantifying the number of antibodies (or other ligands) bound to EVs, and reporting fluorescent trigger thresholds in molecules of equivalent soluble fluorophore (MESF), rather than arbitrary units potentially provides a standardized way of EV detection using fluorescent threshold triggering, allowing readers to determine if it is likely the detected particles were EVs, and method of reporting flow cytometer settings that can be reproduced. Implementing these methods currently pose challenges due to the accuracy and availability of dim fluorescent standards, which will likely be overcome in the near future, with researchers either making their own standards or approximating MESF values with available standards (Stoner et al., 2016). The use of fluorescent standardization for EV analysis using flow cytometry, like scatter standardization, is also yet to be validated.

## Single EV detection

In the case of both scatter and fluorescent threshold triggers, it is important to have reassurance that single particles are being analyzed. Due to most conventional flow cytometers being developed for cellular analysis, the fluidic stream suspending particles have diameters >5 μm with laser beam heights ~10 μm, creating an illuminated cylinder volume of ~200 μm (Colombo et al., 2014). In comparison, a 100 nm EV has a volume of 5.2 × 10^−4^ μm^3^ meaning that if particles are run through a flow cytometer at too high a concentration, it is possible for hundreds of particles to be present at once in the illuminated region of the fluidics stream. A flow cytometer detects these as one large particle, rather than hundreds of individual small particles, as these particle's scatter and fluorescence data from may particles are merged into a single electronic event. This phenomenon has become known as “swarm detection” (van der Pol et al., 2012b). Importantly, this effect can cause particles that are below a trigger threshold, and therefore undetectable, to be detected by the cumulative scatter/fluorescence of many particles present, enabling them to reach the trigger threshold (van der Pol et al., 2012b). This effect therefore distorts the recorded data's concentration and phenotype making it inaccurate, and has resulted in the requirement for control methods to provide reassurance that samples are dilute enough during analysis to ensure single particle analysis. One simple method for demonstrating single particle detection involves analyzing serial dilutions of a sample (van der Vlist et al., 2012; Nolan and Stoner, 2013; Kormelink et al., 2015). If single particles are being detected, the number positive event will halve when serially diluting the samples, forming a linear decline between dilution factor and particle count. Furthermore, the fluorescence intensity of positively stained particles should also remain consistent (Nolan, 2015; Stoner et al., 2016).

## Conclusions and perspectives

Due to the current lack of a gold-standard, the best detection equipment for a study of EVs is dictated by the EVs of interest and type of data to be collected. While current conventional flow cytometers are not yet capable of detecting either single-fluorescent molecules or 30 nm EVs by scatter detection, dedicated flow cytometers can detect 29 nm silica particle scatter above the noise, and single-fluorescent molecules (Hercher et al., 1979; Zhu et al., 2014). This type of dedicated flow cytometry therefore has the potential of detecting and phenotyping the full range of EVs, with commercial flow cytometers capable of this level of resolution likely to be appearing very soon (Hercher et al., 1979; Zhu et al., 2014). Methods of scatter and fluorescent standardization need to be put in place and validated to ensure reliability of published data when comparing data regardless of whether a dedicated or conventional flow cytometer has been used. However, moving forward researchers would enable greater clarity in their EV analysis using flow cytometry by producing serial dilution curves of their EV data, using appropriate MESF standards on the fluorescent channels, and reporting methods to MIFlowCyt guidelines which outline flow cytometer settings and reagents used (Lee et al., 2008).

## Author contributions

JAW organized and prepared this manuscript; JAW, JSW, JH, and NE all contributed to writing and reviewing the major part of the manuscript.

### Conflict of interest statement

NE has received funding and holds a research collaboration agreement with ThermoFisher Scientific. The other authors declare that the research was conducted in the absence of any commercial or financial relationships that could be construed as a potential conflict of interest.

## Abbreviations

dFCM: dedicated small particle flow cytometry
cFCM: conventional flow cytometry
AFM: atomic force microscopy
EM: electron microscopy
SRM: super resolution microscopy
RPS: resistive pulse sensing
DLS: dynamic light scatter
NTA: nanoparticle tracking analysis.
